# Molecular Signatures of Immunity and Immunogenicity in Infection and Vaccination

**DOI:** 10.3389/fimmu.2017.01563

**Published:** 2017-11-15

**Authors:** Mariëlle C. Haks, Barbara Bottazzi, Valentina Cecchinato, Corinne De Gregorio, Giuseppe Del Giudice, Stefan H. E. Kaufmann, Antonio Lanzavecchia, David J. M. Lewis, Jeroen Maertzdorf, Alberto Mantovani, Federica Sallusto, Marina Sironi, Mariagrazia Uguccioni, Tom H. M. Ottenhoff

**Affiliations:** ^1^Department of Infectious Diseases, Leiden University Medical Center, Leiden, Netherlands; ^2^Humanitas Clinical and Research Center, Rozzano, Italy; ^3^Institute for Research in Biomedicine, Università della Svizzera italiana, Bellinzona, Switzerland; ^4^GSK Vaccines, Siena, Italy; ^5^Department of Immunology, Max Planck Institute for Infection Biology, Berlin, Germany; ^6^University of Surrey, Guildford, United Kingdom; ^7^Humanitas University, Department of Biomedical Sciences, Pieve Emanuele-Milan, Italy; ^8^Institute of Microbiology, ETH Zurich, Zurich, Switzerland

**Keywords:** vaccines, biomarkers, immunity and infections, immunity, assays

## Abstract

Vaccinology aims to understand what factors drive vaccine-induced immunity and protection. For many vaccines, however, the mechanisms underlying immunity and protection remain incompletely characterized at best, and except for neutralizing antibodies induced by viral vaccines, few correlates of protection exist. Recent omics and systems biology big data platforms have yielded valuable insights in these areas, particularly for viral vaccines, but in the case of more complex vaccines against bacterial infectious diseases, understanding is fragmented and limited. To fill this gap, the EC supported ADITEC project (http://www.aditecproject.eu/; http://stm.sciencemag.org/content/4/128/128cm4.full) featured a work package on “Molecular signatures of immunity and immunogenicity,” aimed to identify key molecular mechanisms of innate and adaptive immunity during effector and memory stages of immune responses following vaccination. Specifically, technologies were developed to assess the human immune response to vaccination and infection at the level of the transcriptomic and proteomic response, T-cell and B-cell memory formation, cellular trafficking, and key molecular pathways of innate immunity, with emphasis on underlying mechanisms of protective immunity. This work intersected with other efforts in the ADITEC project. This review summarizes the main achievements of the work package.

## Introduction

One of the key goals in vaccinology is to understand what factors drive protective immunity induced by vaccines. In many cases, the precise mechanisms underlying such immunity remain unknown, or at best incompletely characterized. Except for the case of neutralizing antibodies induced by viral vaccines, very few correlates of protection exist. The availability of omics technologies and advanced systems biology big data analytical platforms has enabled large scale analyses of vaccine-induced responses. These efforts revealed novel insights into immunity and correlates of protection, for example, in the human response to yellow fever and influenza vaccination (1, 2). However, for more complex vaccines such as those against bacterial infectious diseases, such understanding as yet is mostly lacking.

To fill this gap, EC FP7 supported ADITEC innovative project featured a work package on “Molecular signatures of immunity and immunogenicity.” The working group aimed to identify key molecular mechanisms of innate and adaptive immunity during effector and memory stages of immune responses following vaccination. Specifically, technologies were developed to assess the human immune response to vaccination and infection at the level of the transcriptomic and proteomic response, T-cell and B-cell memory formation, cellular trafficking, and key molecular pathways of innate immunity, with emphasis on underlying mechanisms of protective immunity. This work was integrated with efforts in other sections of the ADITEC project, such as a work package on immunity and vaccine-induced responses in early life and aging; and work packages on the human response to adjuvants in clinical and translational vaccinology.

The following specific issues will be addressed in detail below:

1. An extended multiplex transcriptomic profiling assay [dual-color Reverse-Transcription Multiplex Ligation-dependent Probe Amplification (dcRT-MLPA)] was developed for bioprofiling the human immune response following vaccination against human infectious diseases, with particular emphasis on transcriptomic signatures of innate, adaptive, regulatory, inflammatory, and memory responses. Determination of optimal responses and dense kinetic transcriptomic response measurements will be helpful in optimizing novel immunization strategies.
2. This platform was complemented by global transcriptomic gene expression profiling (mRNA as well as micro-RNA) following vaccination with a complex vaccine, the live recombinant bacille Calmette–Guérin (BCG) (VPM1002), which had been tested in infants and adults. Transcriptomic analyses were used to dissect novel mechanisms and correlates of immunity and safety, with particular emphasis on age and temporal changes following vaccination.
3. Complementing the work on transcriptomics, quantitative proteomic profiles were determined using novel platform technologies, including hydrogen deuterium exchange (HDX) coupled to mass spectrometry (MS). HDX–MS was used to define functionally active epitopes and antibodies in polyclonal sera following vaccination. Complementing studies addressed the role of T follicular helper (Tfh) cells and explored their presence in the blood as possible biomarkers of protective vaccine efficacy.
4. A key feature of vaccine-induced immunity is the formation of antigen-specific memory T-cells and B-cells, and their ability to migrate to the correct tissue sites. New technology platforms to analyze human B-cell and T-cell immune repertoires, suitable to interrogate large number of B-cells or T-cells for the first time, allowed a comprehensive analysis of the memory response in all its cellular components. A combination of experimental approaches (antigenic stimulation, TCR deep sequencing and cloning of Th1, Th2, and Th17 memory subsets) allowed the dissection of T-cell subset responses, showing that pathogen- or vaccine-induced T-cells are functionally heterogeneous and comprise both clones polarized toward a single fate, as well as clones whose progeny has acquired multiple fates. By immortalizing memory B-cells from donors upon influenza vaccination, a new type of rarely occurring influenza-neutralizing antibodies targeting a conserved site in the hemagglutinin (HA) stem was found.
5. To dissect the molecular mechanisms regulating T-cell trafficking at mucosal sites in health and disease, T-cell migration in response to chemokines CCL20, CXCL10, and CXCL12 was assessed. T-cell migration in HIV-1 infection was evaluated as a model of impaired migration to mucosal sites, as it is well known that antiretroviral therapy (ART) therapy is not able to fully restore complete T-cell repopulation of the intestinal mucosa in HIV-1 infected patients. Both CCR6+ and CXCR3+ CD4+ T-cells from HIV-1 infected patients, albeit expressing the same number of chemokine receptor as in healthy individuals, were characterized by an inefficient polymerization of actin after receptor triggering, regardless of ART therapy. This impairment was also confirmed in a macaque SIV infection model. *In vivo* studies showed that persistent immune activation, but not the presence of the virus, was responsible for the alterations in the cytoskeleton machinery, resulting in altered cellular trafficking to the mucosal compartment. This could be redressed by pharmacological intervention, restoring effective lymphocyte migration. These results highlight the importance of evaluating the capability of Th cells to reach mucosal niches to support cell maturation and functional activity, particularly in individuals experiencing chronic immune activation, and thus suggest new molecular strategies for improved vaccination.
6. Activation of innate immunity is key to adjuvant activity and expression of vaccine effector mechanisms. Since plasticity and polarization are key components of innate immunity, key molecular markers of innate immunity were investigated in the context of adjuvant activity and expression of vaccine effector mechanisms. One focus was on the role of pentraxin 3 (PTX3), a member of the pentraxin family involved in innate resistance, as an endogenous adjuvant in vaccination using outer membrane vesicles (OMV) from *Neisseria meningitidis* (*Nm*) as a model vaccine. In addition, the role of PTX3 in innate resistance to pathogens, particularly in urinary tract infections was assessed. This work allowed the description of the association of *PTX3* human genetic polymorphisms with susceptibility to infections with *Aspergillus fumigatus* in immunocompromised patients and with uropathogenic *Escherichia coli*. In addition, the role of PTX3 in tissue remodeling was defined and novel markers associated with macrophage polarization discovered.

Each of these topics will be discussed in more detail, followed by a concluding section.

## A dcRT-MLPA Assay to Identify Novel Diagnostic, Prognostic, and Predictive Biomarkers in Response to Vaccination, Infection, Active Disease, and Cure

Dissecting innate, adaptive, and inflammatory immune signatures underlie the identification of biomarker signatures that can be used to diagnose infection or disease; to predict progression toward disease in the infected host; to monitor the efficacy of disease treatment; or to predict (in)adequate responsiveness to novel or existing vaccines. The latter signatures will greatly aid the rational development, testing and evaluation of novel vaccines. They may also allow the study of the impact of host factors such as age (elderly vs. children), coinfection [HIV, CMV, Epstein–Barr virus (EBV), etc.], comorbidity (type 2 diabetes, allergic, and inflammatory disorders) or iatrogenic immunosuppression (biologicals, other immunosuppressants) on responses to vaccines.

To investigate such human immune responses at the transcriptomic level, we have developed a dcRT-MLPA assay (3). dcRT-MLPA is a focused gene expression profiling platform that has proven suitable to identify, monitor, and validate multicomponent host diagnostic and prognostic/predictive biomarker signatures in many human cohort studies (4). The studies described in this article focus on the discovery of diagnostic, prognostic, and therapy-responsive biomarker signatures mainly in tuberculosis (TB). Genes were selected for inclusion in the dcRT-MLPA targeted gene expression profiling platform based on their ability to assess various compartments of the human immune response, with key roles in inducing and skewing immune reactivity and inflammation. This included genes representing adaptive immune responses (T/NK/B-cell, Treg, and Th1/Th2/Th9/Th17 and Th22 responses, cytotoxicity, and cell subset markers); innate immune responses (myeloid associated markers and scavenger receptors, pattern recognition receptors, and inflammasome components); inflammatory and IFN-signaling genes; and genes associated with apoptosis, cell growth/proliferation, transcriptional regulators/activators, mitochondrial stress, and inflammation.

### Diagnostic Biomarkers

Tuberculosis is a global health problem and the world’s leading cause of death from infectious diseases [World Health (5)]. One-fourth of the worldwide population is latently infected with *Mycobacterium tuberculosis* (*Mtb*), the pathogen causing TB, while active disease develops in about 5–10% of infected individuals. Understanding the differences in immune responses between individuals able to control the infection versus those who develop active disease, preferably in a preclinical stage, would greatly facilitate vaccine development. However, since no correlates of protection have been uncovered yet and vaccination with BCG mainly contributes to the prevention and control of childhood but not adult TB, effective control of the TB pandemic still mainly depends on accurate diagnosis and early detection of active disease, allowing curative treatment to be initiated before TB transmission can occur. Because current immunological tools to diagnose TB infection, such as the tuberculin skin test and interferon-gamma release assays are not able to distinguish between latent TB infection (LTBI) and active TB disease, and have poor sensitivity in children and immune-compromised individuals, direct *ex vivo* whole blood RNA expression profiling was performed in genetically and geographically diverse adult, pediatric and HIV-infected cohorts to identify biomarkers that can classify clinical stages of TB independent of age, HIV status and genetic background.

Characterization of the human immune response to *Mtb* in cohorts from South Africa, Malawi, The Gambia, Ethiopia, India, and Paraguay identified biomarker signatures that were strongly associated with active TB and were strikingly distinct from that associated with latent infection or uninfected controls (3, 6–10). Although the compiled multicomponent signatures differed to a certain extent between study cohorts from different geographic origins, the most robust single classifier discriminating between active disease and latent infection or uninfected controls was *FCGR1A* [high-affinity IgG Fc receptor 1A (CD64)]. This marker is significantly higher expressed in individuals with active TB than in those with LTBI regardless of age (8, 10), HIV status (7, 9), or geo-genetic differences (3, 6, 7). This demonstrates the power of this biomarker to classify the different clinical outcomes of exposure to *Mtb* and warrants further analysis of the role of FCGR1A in TB pathogenesis.

### Biomarkers of the Curative Response to Therapy

Multi-, extensive-, and total-drug resistant TB (MDR/XDR/TDR-TB) continues to emerge and is primarily caused by the use of ineffective formulations of drugs (such as use of single drugs) and poor adherence by TB patients to the strict 6-month drug treatment regimen. Therefore, monitoring early kinetics in treatment responses is key to the effective delivery of anti-TB treatment (ATT) and to prevent *de novo* drug resistance.

Longitudinal follow up of TB cases during ATT showed that biomarkers that can discriminate active TB from LTBI and uninfected controls can also be used to monitor TB treatment responses independent of age, HIV status, or genetic background (3, 8; Gebremicael et al., forthcoming^1^). In immune-competent adults from The Gambia, gene expression profiles normalized over time and were similar to those observed in LTBIs and uninfected controls at the end of treatment (6 months) (3). However, changes in gene expression levels during ATT showed distinct kinetics. The expression levels of a proportion of genes normalized to the expression levels observed in LTBIs within 2 months (e.g., *BLR1*), whereas others reached control levels only after the full 6 months of treatment (e.g., *FCGR1A*). Importantly, in HIV-coinfected TB patients, transcriptomic profiles of ATT treatment responses (including expression of *FCGR1A*) were identified that were not affected by highly active antiretroviral therapy, and that normalized to levels observed in HIV+ latently TB infected and uninfected controls after completion of ATT (see text footnote 1). Moreover, in an Indian pediatric cohort, baseline levels of *BLR1* and *FCGR1A* displayed a capacity of >70% to predict the six month treatment outcome (8). Together, these data suggest that *FCGR1A* could possibly serve as part of a reliable and robust predictive signature of the treatment response, and as such help optimizing personalized medicine in TB while minimizing *de novo* drug resistance, independent of age, immune status and genetic background.

### Prognostic Biomarkers

Because most immunocompetent individuals maintain a lifelong latent infection with *Mtb* and active disease develops in only a minority of infected individuals, identification of correlates of TB progression would substantially contribute to combat the TB epidemic. In TB endemic areas, such biomarker signatures could identify individuals at risk of developing TB, allowing curative treatment to be initiated before TB transmission can occur.

Transcriptomic profiling of the immune responses of immune-competent adult progressors versus non-TB progressors in a well-characterized TB case-contact platform from The Gambia identified—among others—antiapoptotic gene *BCL2* as a marker that might predict the onset of active disease very early after infection (11). *BCL2* was significantly lower expressed in progressors compared with non-progressors, and dysregulation of *BCL2* occurred as early as 1 year before progression toward TB disease. Corroborating these findings at a cellular level, increased levels of apoptosis in effector T-cells were found to constitute a risk factor for TB disease progression (12).

In addition, in a retrospective case–control study in The Netherlands of a high risk population of HIV-infected drug users, *IL13* and *AIRE* were identified as markers correlating with progression to TB, months before clinical diagnosis (13). Furthermore, transcriptomic profiles of *IL13*-expressing TB cases were strongly enriched for type I IFN related signaling genes, suggesting that these genes represent processes that contribute to TB pathogenesis in HIV-infected individuals. Although the association between *IL-13-AIRE* and TB progression could not be validated in independent cohorts of HIV-negative South African and Gambian adult progressors and controls, activation of the interferon response, however, was validated as a signature predictive of progression toward active disease (14). Using RNA-Seq, a whole blood transcriptomic mRNA expression signature was identified in a large prospective cohort of LTBI adolescents based on the differential expression of 16 human genes (14). Interestingly, also *FCGR1A* (as well as *FCGR1B*) was again part of the predictive signature, in agreement with the importance of these and other IFN-regulated genes in TB pathogenesis.

Once these biomarker signatures have been validated in larger cohorts, they will provide an initial platform to improve diagnosis of TB, monitor TB therapeutic interventions and prospectively identify people at risk of developing TB. More importantly, these biomarker signatures may help unravel and improve our understanding of protective immunity to TB, which is a prerequisite for the development of an effective TB vaccine.

## Transcriptome Profiles in Infants Vaccinated with Recombinant BCG VPM1002

The current TB vaccine *Mycobacterium bovis* BCG prevents severe disseminated TB in children, but fails to protect against pulmonary TB. The recombinant BCG ΔureC:hly (VPM1002) was designed to improve safety and immunogenicity (15). This recombinant vaccine was modified to express the membrane-perturbing listeriolysin (Hly) of *Listeria monocytogenes*, allowing egression of mycobacterial antigens into the cytosol for better antigen presentation. Simultaneously, its urease C deficiency prevents VPM1002 from neutralizing the acidic pH inside the phagosome, which is required for the biological activity of Hly (16).

*In vitro* and animal studies have indicated an improved cross-priming and increased apoptosis in VPM1002 infected macrophages, resulting in improved vaccine efficacy over parental BCG (15, 16). Superior protection by VPM1002 was later shown to relate to earlier recruitment of type 1 cytokine producing T-cells and a profound capacity to produce type 17 cytokine responses, which was not seen after BCG vaccination (17). Moreover, VPM1002 stimulates enhanced AIM2 inflammasome activation, enhancing autophagy and secretion of (IL)-1β and IL-18 (18). Experiments with immunodeficient mice revealed higher safety of VPM1002 as compared with parental BCG. Enhanced safety of VPM1002 is also illustrated by a lower incidence of abscess formation in immunized infants (19).

VPM1002 has now completed phase I clinical trials in healthy adults (19, 20) and a phase IIa trial in South African newborns. It is currently undergoing phase II trial assessment in HIV-exposed neonates (NCT01479972). Within the ADITEC project we have generated gene expression profiles from infants participating in the phase IIa trial. Two groups of 11 neonates each receiving either VPM1002 or parental BCG were included, and gene expression profiles were analyzed from samples taken at the time of immunization and at weeks 2, 6, 12, 18, and 26 postvaccination. Safety and immunogenicity data from this clinical trial revealed that safety parameters for VPM1002 and the parental BCG strain are comparable. Both vaccines induced IFNγ responses, while VPM1002 vaccination in addition resulted in an increased proportion of IL-17 producing CD8 T-cells (19).

**Figure 1:**
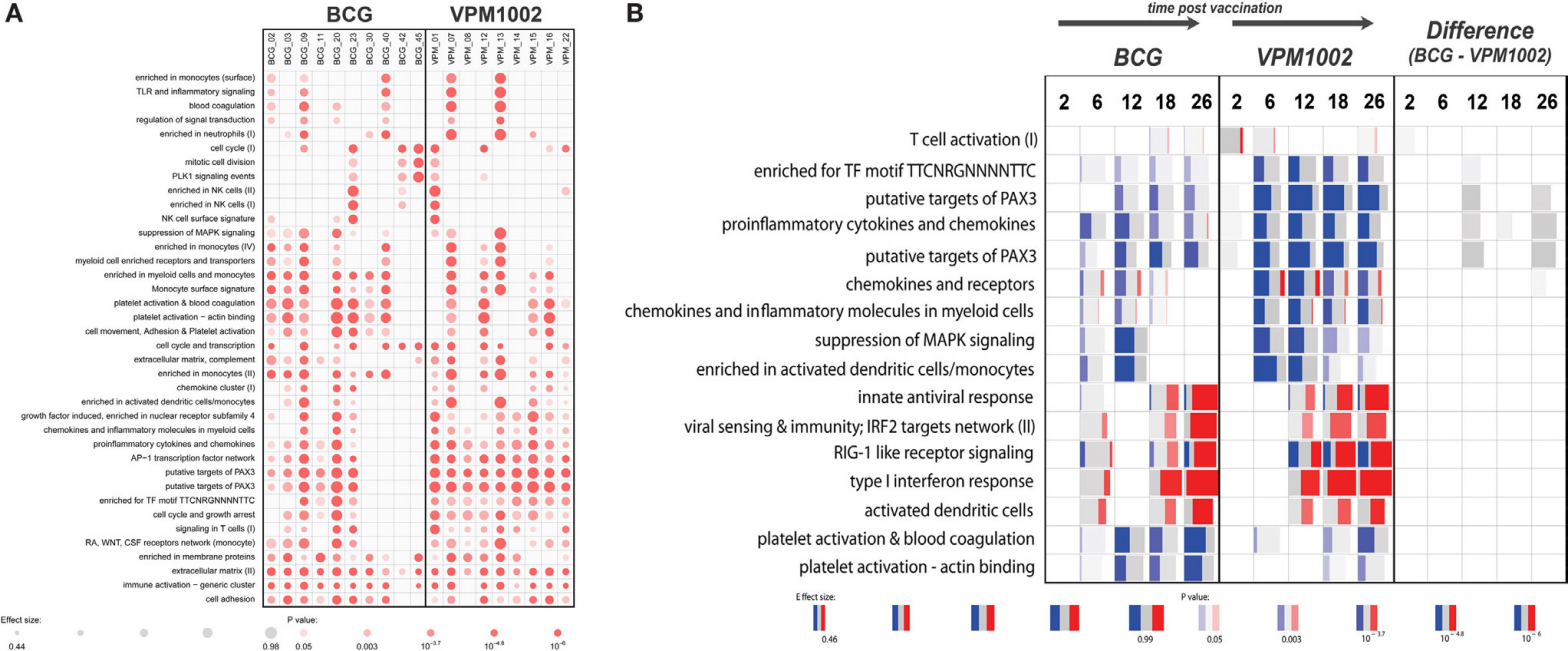
Gene set enrichment results generated with the tmod R package. **(A)** Enrichment in gene modules in each individual at 26 weeks postvaccination compared with baseline. **(B)** Gene set enrichment in both groups and between the groups at different time points compared with baseline. Red and blue indicate the proportion of genes in a particular module that is upregulated or downregulated respectively. Width of each box relates to its effect size, while brighter colors indicate lower *p*-values. Panel **(B)** represents a selection of a larger number of enriched gene modules.

The gene expression profiles that were generated showed a substantial variability in responses between individuals (Figure 1A). Following vaccination early after birth, dramatic changes in the gene expression profiles over time were observed in both groups. The most pronounced changes were associated with upregulation of immunoglobulin-related genes, which likely reflects the maturation of the immune system in infants. In a direct comparison between the groups vaccinated with parental BCG and VPM1002, we did not observe any significant differences in gene expression at any of the time points analyzed. Most likely, differences in gene expression induced by the two vaccines, if any, are too small to be detected and may be obscured by the pronounced changes over time. Second, this study was set up as a safety trial with a limited number of individuals. As a consequence, the gene expression analysis was statistically underpowered.

Therefore, we harnessed the power of gene modules, using a new bioinformatics tool called “tmod” (J. Weiner https://cran.r-project.org/web/packages/tmod/index.html) to calculate enrichments in genes associated with particular biological responses. Results from this analysis indicated subtle functional differences between the vaccination groups that could not be detected at the single gene level (Figure 1B). For example, the VPM1002 vaccinated group showed earlier and more pronounced changes in genes involved in T-cell activation. Similarly, a prolonged enrichment in myeloid/monocyte-associated genes could be observed in the VPM1002 group (Figure 1B). In a direct comparison between the groups, we did observe a weak enrichment suggesting a slightly stronger T-cell activation in the first weeks following VPM1002 immunization. Also, a more pronounced downregulation of *PAX3* target genes and pro-inflammatory cytokine and chemokine expression in the VPM1002 group, although none of these enrichments were statistically significant (Figure 1B).

## Novel Technologies for Epitope Mapping for Vaccine Design, and Exploration of Tfh Cells as Novel Biomarkers

The analysis of the B-cell repertoire could not be considered complete if the fully sequenced IgG are not functionally characterized, quantified in the serum, and the epitopes recognized are not defined. All this provides essential information that can be used to guide vaccine design and optimization, eventually to predict vaccination efficacy and its duration. Considering that the majority of the functional B-cell epitopes are discontinuous non-linear epitopes having 3D-conformational structures, it is important to apply methodologies able to decipher these sometimes complex structures.

We used HDX coupled to MS to allow the mapping of conformational epitopes (21–27). The approach was also compared with the most sophisticated and available approaches such as protein chip (26, 27), phage display (21, 26, 27), X-ray (21), and cryo-EM (24, 25). Modern HDX–MS is more straightforward, rapid, and routine than in the past. As a result, the breadth of applications of the method, including epitope mapping, has expanded (28). HDX–MS relies upon the rapid exchange of backbone amide hydrogen for deuterium when a protein is diluted into a deuterated buffer. Each exchange event increases the protein mass of 1 Da that can be monitored by MS. Epitope mapping through HDX is based on differential rates of deuterium incorporation by the antigen when it is bound or not with a specific mAb (29). When an antigen–antibody complex is formed, the interface between the two components can occlude solvent, thereby reducing the exchange rate due to the action of solvent steric exclusion. Deuterium incorporation of the free antigen and of the antigen–mAb complex is compared and antigen sequences that present reduced exchange kinetics when the antigen is bound to mAb are highlighted as potential epitopes.

In a first phase, we probed the binding between mAb 12C1 and fHbp, using hybrid approaches including peptide arrays, X-ray crystallography, phage display, and HDX–MS, evidencing that HDX–MS, although not the highest resolution method, is the most effective in providing nearly complete information about the structure of epitopes (21). The approach has sufficient resolution to recognize fHbp overlapping epitopes with different functional properties (22). It was also successfully applied to map epitopes from NadA (23, 26) and NHBA (27), the two other protective epitopes of the vaccine against group B meningococcus, Bexsero. Hybrid approaches making use of HDX–MS and electron microscopy also evidenced the power of HDX–MS to map viral antigen epitopes of CMV gH–gL complex. In addition, to better characterizing the immune response to vaccination and to support antigen design, this method that speeds up the elucidation of recognized epitope will contribute to increase the number of available structures of antigens and antigen–antibody complexes, opening new possibilities for the development of novel tools that might reliably predict protective epitopes.

## Influenza-Specific Circulating T-Cell Subsets as Early Predictors of Specific Antibody Responses

Development of vaccines is a long endeavor requiring long and expensive clinical trials to prove the efficacy of vaccines in the relevant target populations. The availability of biomarkers predicting protection or predicting protective immune responsiveness may accelerate the development of vaccines.

T follicular helper cells are a CD4+ T-cell subpopulation that is identifiable in lymph nodes and tonsils. Tfh cells are specialized in providing help to B-cells (30–32). The identification of a circulating counterpart of the Tfh subset in the blood (33, 34) would allow the measurement of these cells following vaccination, which may help defining markers predicting vaccine efficacy. Therefore, the question was to investigate whether IL-21+ CD4+ T-cells induced by specific vaccination were detectable in human blood, if and how vaccination modulated their frequency, and whether their expansion correlated with increased titers of functional antibodies. Indeed, following vaccination with MF59-adjuvanted avian H5N1 vaccine, H5N1-specific IL-21+ CD4+ T-cells were detectable in the blood, expanded after vaccination and accumulated in the CXCR5−ICOS1+ subset. The rise of vaccine-specific ICOS1+ IL-21+ CD4+ T-cells appeared to predict the postvaccination increase of functional antibodies in these vaccines. Finally, circulating CXCR5−ICOS1+ CD4+ T-cells contained increased numbers of T-cells able to help influenza-specific B-cell differentiation, such that they differentiated *in vitro* into antibody-secreting cells in a manner that was dependent on IL-21− and ICOS1 (35). More recent studies have confirmed these findings and have also shown that the early expansion of cells with a Tfh phenotype predicts the long term persistence of neutralizing antibodies against influenza virus (36). Thus, the expansion of antigen-specific ICOS1+ IL-21+ CD4+ T-cells in the circulation may represent an early predictor of a vaccine’s ability to stimulate vaccine-specific immunity and a useful surrogate marker of a vaccine’s immunogenicity in human beings.

## Heterogeneity of Memory CD4+ T-Cells Induced by Pathogens or Vaccines

CD4+ T helper cells are crucial players in the adaptive immune response, contributing to protection against a wide range of pathogens through the functional regulation of other immune and non-immune cell types. CD4+ T helper cells are characterized by functional diversity that has evolved to provide the most appropriate type of response against different classes of pathogens in different tissues (37). Th1 cells produce IFNγ, express the transcription factor T-bet and, through the activation of macrophages, contribute to immunity against intracellular pathogens. Th2 cells produce IL-4, IL-5, and IL-13, express the transcription factor GATA-3 and, through the activation of eosinophils and mast cells, contribute to protection against helminth parasites. Th17 cells produce IL-17A, IL-17F, and IL-22, express RORγt and together with neutrophils, mediate protection against fungi and extracellular bacteria. Th1, Th2, and Th17 cells differ also in the expression of chemokine receptors that control their homing ability, a property that is coordinately obtained during the process of T-cell differentiation (38). CCR5 and CXCR3 are expressed by Th1 cells, CCR4, CCR3, and CRTh2 by Th2 cells, and CCR6 and CCR4 by Th17 cells. While the role of distinct subsets of CD4+ T-cells in protection against different types of pathogens has long been recognized, it has been unclear whether a given vaccine or pathogen will induce a single type of T-cells and whether a single naive T-cell challenged by a pathogen or by a vaccine may acquire multiple T-cell fates.

**Figure 2:**
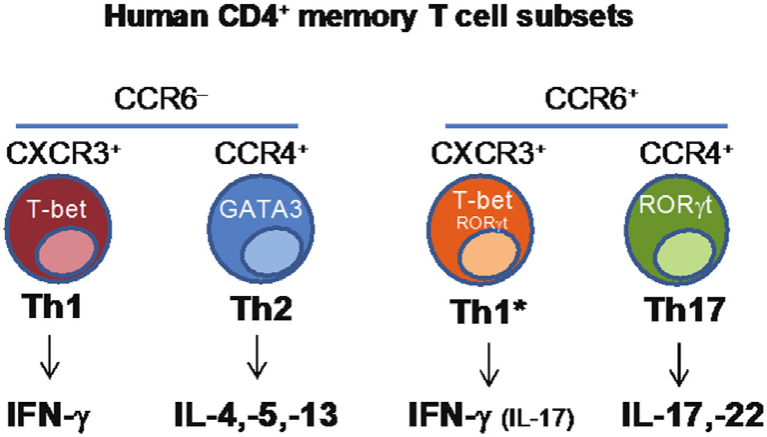
Identification of human CD4+ memory T-cell subsets based on the differential expression of chemokine receptors.

We combined antigenic stimulation, TCR deep sequencing and cloning of human Th1, Th2, and Th17 memory subsets, to study the distribution and TCR repertoire of pathogen- and vaccine-specific T-cells in immune donors (39). We found that memory T-cells induced by *Candida albicans* are present at high frequency in a CCR6+ compartment, which comprises Th17 and a subpopulation of Th1 cells co-expressing T-bet and RORγt (defined as Th1*), and at low frequency in a CCR6− compartment, which comprises classical Th1 and Th2 cells (40) (Figure 2). We could then demonstrate, using next-generation TCR Vβ sequencing, that multiple different clonotypes were present in more than one subset and, in several cases even in all subsets, whereas by contrast other clonotypes were found only in one particular subset. We also studied tetanus toxoid (given with alum as adjuvant) vaccine-induced memory T-cells. Also in this case, TCR Vβ sequencing revealed a high level of clonotype sharing among Th1, Th2, Th1*, and Th17 subsets, with multiple different clonotypes represented in three and even all four subsets. By contrast, *Mtb*-specific memory T-cells in healthy donors were highly enriched in the Th1* subsets, with some specific T-cells present in the Th17 subset. In contrast to what was observed for *C. albicans*, however, only very few clonotypes were shared between *Mtb*-specific Th1* and Th17. Taken together, our results indicate that human T-cells induced by pathogens or vaccines are functionally heterogeneous and comprise both clones polarized toward a single fate, as well as clones whose progeny has acquired multiple fates. The highly significant intraclonal heterogeneity observed in this work further supports the one cell-multiple fates model of CD4 T-cell differentiation; furthermore these data reveal T-cell plasticity in the context of the human T-cell immune response. An important question that this study raises is whether the induction of functionally diverse T-cell subsets by for instance a vaccine is advantageous for the host. Clearly, defective induction of the correct type of Th cell response may increase susceptibility to infections, while the induction of a wider spectrum of different T-cell types, including effector and memory T-cells with different migratory abilities would enlarge the range of differentiated precursors that the host could recruit and expand whenever necessary.

## A New Class of Rare Influenza-Neutralizing Antibodies Target a Conserved Site in the HA Stem

By immortalizing memory B-cells from donors upon influenza vaccination, a new type of rarely occurring influenza-neutralizing antibodies, targeting a conserved site in the HA stem was found.

High throughput cellular screens have been developed and used to isolate potently and broadly neutralizing antibodies against a plethora of pathogens. We used EBV in the presence of CpG to immortalize memory B-cells with high efficiency, and screened culture supernatants for the presence of the desired antibodies using multiple assays based on binding or viral neutralization. Using this approach, we studied the antibody response to influenza HA. The neutralizing antibody response is dominated by antibodies binding the most variable part of the HA, the globular head, which is undergoing antigenic drift continuously. As a consequence, most of anti-head antibodies, which are detected using the classical hemagglutination inhibition assay, neutralize only a few isolates within a given subtype. We and others have previously described heterosubtypic neutralizing antibodies that can bind and neutralize different influenza virus subtypes, comprising group 1 viruses and even group 1 and group 2 viruses (41). The broadly neutralizing antibodies target a conserved site in the HA stem and use a particular VH gene, VH1-69. These antibodies are highly mutated, and they are produced by some but not all individuals. This would suggest that a series of somatic mutations may be necessary for the development of such antibodies. To address the developmental pathway of anti-stem antibodies, we isolated, from a single donor, 197 VH1-69+ anti-stem antibodies. Using sequence information, we performed a genealogical analysis to reconstruct the developmental pathways of a number of the VH1-69 clones. We identified key elements which were required for affinity maturation. In all cases tested, the binding to HA was exclusively dependent on the mutated VH, while the light chain did not play a significant role since it could be substituted by irrelevant L chains. Strikingly, in most clones, affinity maturation was achieved through a single somatic mutation leading to a proline to alanine substitution at position 52a in HCDR2. This mutation was the first to occur in the B-cell clone and was sufficient to confer high-affinity binding to the selecting H1 antigen. This observation is consistent with rapid affinity maturation. Unexpectedly, however, further favorable mutations continued to accumulate, which further increased the breadth of antibody reactivity while making both the initial mutations functionally redundant. We also determined that phenylalanine 54, which was characteristic of all antibodies, was an essential binding residue. Interestingly, this position is polymorphic and individuals lacking a phenylalanine 54 allele did not produce VH1-69 antibodies to the HA stem. Finally, all VH1-69 anti-stem antibodies shared a 13-aminoacid-long HCDR3 with a tyrosine at position 98. This study defines VH1-69 polymorphism, HCDR3 sequence constraints and an individual somatic mutation as the three requirements involved in the generation of broadly neutralizing antibodies. It also reveals an hitherto unknown and unexpected redundancy in process of affinity maturation (42).

## Molecular Mechanisms Regulating T-Cell Trafficking at Mucosal Sites in Health and Disease: Chronic Immune Activation Dampens Leukocyte Trafficking and Calls for Novel Vaccination Strategies

The homing of leukocytes in general, and antigen-specific T-cells in particular, to peripheral tissues, mucosal sites, and secondary lymphoid organs is controlled, among others, by the local production of chemokines, the expression of chemokine receptors on the cell surface, and an efficient cytoskeleton machinery (43, 44). The characterization of the surface expression of the different chemokine receptors on T-cells has guided the discovery of different T helper cell subsets and is still a precious tool for the characterization of novel functional subsets. Nevertheless, scanty information is available on the influence that pathological conditions can have on the activity of chemokine receptors, even if their expression on the T-cell surface remains unaltered compared with healthy individuals. In health and disease, the microenvironment can further control cell migration, by releasing factors that cooperate with chemokines for enhancing cell responses (45–49), or by producing natural chemokine antagonists that block chemokine-induced activities (49). In addition, to the proteins produced by the microenvironment, systemic chronic immune activation can dampen T-cell responses to chemokines (50). HIV-1 infection is a clear example for this phenomenon, since chronic immune activation due to microbial translocation has been associated with poor T-cell repopulation of the intestinal mucosa during ART (51, 52). CD4+ T-cells expressing the chemokine receptors CCR6+ and/or CXCR3+ can traffic to the intestine in response to CCL20 and to IFNγ-induced chemokines, to help maintain the integrity of the mucosal barrier (53). In HIV-1 infected patients, regardless of ART therapy, CD4+ T-cells inefficiently migrate after chemokine triggering, due to an unproductive polymerization of actin (50). Interestingly, an *in vivo* model of sustained toll-like receptor (TLR) 7 triggering with R848 recapitulated chronic immune activation, lymphoid system disruption, and poor response to chemokines as observed in HIV-1-mediated pathology (50, 54). It is indeed the persistent immune activation, and not the presence of the virus, that is causing the impaired response to chemokines. This deficiency is due to alterations in the cytoskeleton machinery that could be used as a marker to assess T-cell anergy caused by TLR7 triggering (55). Pharmacological intervention acting on the cytoskeleton can restore a proper response to chemokines both *in vitro* and *in vivo* (50) and represents novel therapeutic approaches, which are also amenable for combination with other therapies such as ART. It will be important to verify indeed whether such new immunotherapeutic interventions, aimed at dampening chronic immune activation during HIV-1 infection (56), result in efficient restoration of leukocyte migration. This is of particular interest since chronic activation of the immune system is not only present in HIV-1 infection, but is also a signature of other persistent infections as well as autoimmunity. Moreover, inflammation is a major component of senescence and aging-associated pathology (57–59). The study of frail cohorts of individuals and patients will identify those in need of receiving novel vaccine formulations that would improve the ability of leukocytes to migrate to the site of vaccination and later also during infection to properly mount an effective immune response.

## Dissecting Innate Immunity and Developing Tools for Innate Immune Bioprofiling

Activation of an innate inflammatory response is a key step in the mechanisms of action of adjuvants. Gene expression profiling of the mouse muscle injected with different adjuvants revealed modulation of a cluster of innate immunity genes, including particularly: cytokines, cytokine receptors, chemokines, adhesion molecules, and proteins involved in host–pathogen interactions (60). In particular the long *PTX3*, a soluble pattern recognition molecule identified in the 1990s (61), was among the over 1,000 genes upregulated by adjuvant treatment. PTX3 is classified as a member of the pentraxins, a family of highly conserved molecules characterized by a unique multimeric structure (62, 63; Garlanda et al., forthcoming^2^). Based on the sequence of the composing promotors, two subfamilies can be distinguished: short and long pentraxins. C-reactive protein (CRP) and serum amyloid P component are prototypic short pentraxins, by contrast PTX3 is a prototypic long pentraxin subfamily member.

Despite their sequence homologies, CRP and PTX3 differ in molecular structure, gene organization and cellular sources. CRP is produced only by the liver in response to IL-6 while PTX3 is produced locally and more rapidly by myeloid and stromal cells in response to primary pro-inflammatory cytokines (IL-1β and TNF-α) and ligation of TLRs. In addition, PTX3 is stored in neutrophils-specific granules, from which it is promptly released upon microbial recognition (63). From a functional point of view, PTX3 is endowed with multifunctional properties at the crossroad of inflammation, innate immunity, tissue remodeling, and female fertility. Of note, PTX3 plays an essential non-redundant role in resistance to selected microbes. The protein has antibody-like properties, binding selected pathogens (i.e., *A. fumigatus, Pseudomonas aeruginosa*, and more) and conserved microbial moieties (outer membrane protein A from *Klebsiella pneumoniae*; HA glycoprotein from influenza virus), and facilitating phagocytosis of recognized microbes in a manner dependent on Fcγ receptors and complement (64, 65). Moreover, PTX3 orchestrates complement activity (66) and, by regulating complement-dependent tumor promoting inflammation, can act as an extrinsic oncosuppressor gene in murine and human tumors (67). Finally, recent results obtained in different models of tissue damage highlighted a non-redundant role of PTX3 in remodeling and repair of tissue *via* its interaction with fibrin (68). This further supports the evidence that the recognition of matrix and microbial components is shared ancestral features of the humoral arm of the innate immune system.

**Figure 3:**
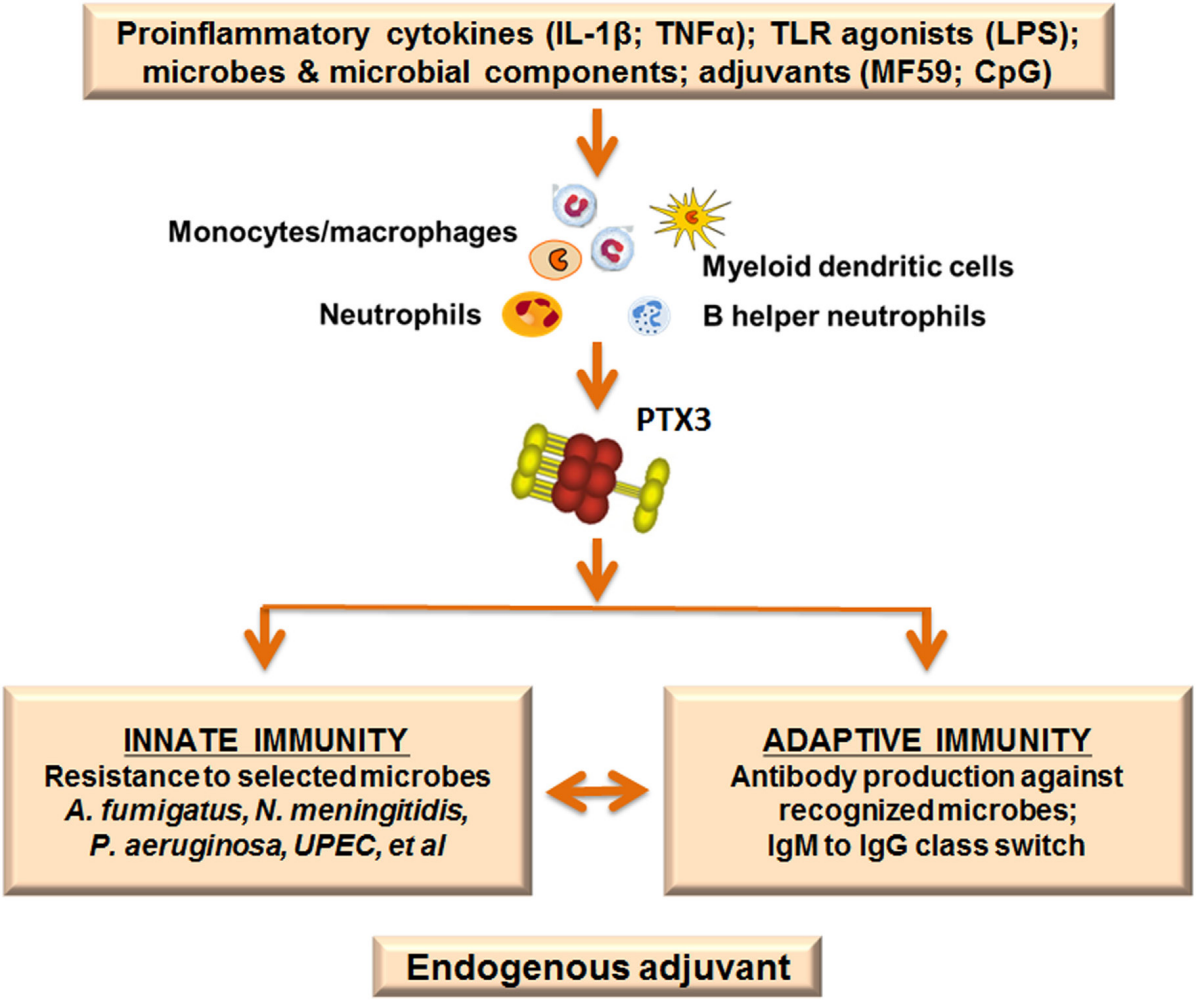
Pentraxin 3 (PTX3) as endogenous adjuvant. PTX3 is locally produced at sites of infection or inflammation by myeloid cells in response to primary pro-inflammatory cytokines and toll-like receptor (TLR) engagement. In addition, the PTX3 gene is rapidly upregulated after treatment with selected adjuvants (MF59; CpG). PTX3 plays essential roles in the immune response to recognized pathogens: it is an active player in the innate immune response to microbes, acting as an opsonin and enhancing phagocytosis. Moreover, PTX3 is involved in immunoglobulin production and class switching from IgM to IgG, promoting humoral adaptive responses. The function of PTX3 as a bridge between the humoral arms of the innate and adaptive immune systems is in agreement with a role as endogenous adjuvant.

In view of the upregulation of PTX3 by selected adjuvants we investigated the role of this protein in the antibody response using a well-known model of vaccination with OMV from *Nm*. We found that PTX3 binds *Nm* as well as OMV derived from *Nm*, exerting a protective role in a model of infection with *Nm* in the infant rat. *Ptx3*-deficient mice vaccinated with OMV without any adjuvant developed a lower antibody response compared with WT mice. In addition, co-injection of PTX3 enhanced the antibody response, especially in *ptx3*-deficient mice. Recognition of the antigen is essential for the effect exerted by PTX3, since immunization with an antigen not recognized by PTX3, such as ovalbumin, induced similar response in WT and *ptx3−/−* mice (69). This observation was confirmed by further investigations showing that *ptx3−/−* mice produced lower levels of IgM in response to administration of Pneumovax, a human vaccine containing capsular polysaccharide from multiple *Streptococcus pneumoniae* (*Sp*) serotypes (70). Similarly, *ptx3−/−* mice produced less antibodies following immunization with PR8 influenza A virus, which is also recognized by PTX3 (71), and in both cases PTX3 administration restored the response. Searching for an explanation of these effects, it has been found that PTX3 was released by B helper neutrophils (NBh) and bound to B-cells located in the marginal zone (MZ) in the spleen. NBh are an innate-like subset of antibody-producing cells positioned at the interface of the adaptive immune system and the circulation. The interaction between PTX3 and MZ-B-cells activated signals that were independent of FcγR but triggered class-switching from IgM to IgG. PTX3 promoted MZ-B-cell differentiation into extrafollicular plasmablasts and plasma cells, and enhanced IgM and IgG responses to the encapsulated bacterium *Sp*, or after immunization with capsular polysaccharides or bacterial carbohydrates. These results indicate that the humoral pattern recognition molecule PTX3, produced in response to pro-inflammatory cytokines or selected adjuvants, may amplify effective adaptive antibody responses that are induced by antigens recognized by PTX3, thus serving as an endogenous adjuvant (Figure 3).

The data outlined above provide a rationale to evaluate whether PTX3 could be a correlate of the shaping of the immune response induced by adjuvants in humans. In collaboration with D. J. Lewis and G. Del Giudice, PTX3 and CRP plasma levels were measured in a cohort of individuals injected with placebo or with licensed influenza vaccines adjuvanted or not. This study received ethical approval from London—Surrey Borders Research Ethics Committee (REC Ref: 13/LO/0044) and was registered on ClinicalTrials.gov before enrollment (NCT01771367). Preliminary unpublished results evidenced an increase in PTX3 plasma levels at early time points in individuals injected with the adjuvanted vaccine, confirming in humans the effects on PTX3 gene upregulation initially observed in mice (60). In addition, after immunization with an adjuvanted vaccine we observed an earlier induction of PTX3 (peak at 24 h postinjection) compared with CRP (peak at 48 h postinjection), likely due to the local expression of PTX3 versus the systemic production of CRP.

Several single nucleotide polymorphisms (SNPs) have been described in the human *PTX3* gene, mainly located in its non-coding regions with the only exception of one exonic SNP causing an amino acid variation in position 48 (Asp48Ala). *PTX3* haplotypes are associated with increased susceptibility to lung TB (72), *P. aeruginosa* infections in cystic fibrosis Caucasian patients (73), *A. fumigatus* infections in bone marrow transplanted patients (74), and urinary tract infections (75). Results in a large cohort of patients with fungal infections after solid organ transplantation (76) and in 2,609 bone marrow transplanted patients and their donor pairs (77) further reinforced the association of *PTX3* gene SNPs and susceptibility to fungal infection. The protection-associated haplotypes were also associated with higher PTX3 protein expression and circulating levels (74, 76), supporting an active role of PTX3 as non-redundant player involved in the innate defense against recognized pathogens.

In summary PTX3, a molecule of the innate immune system, produced in response to pro-inflammatory mediators, not only acts as an antibody-like molecule, recognizing pathogens and promoting their removal but also helps in antibody production by adaptive immunity, acting as endogenous adjuvant. This is schematically depicted in Figure 3.

## Concluding Remarks

Understanding the factors that are critical for the induction of protective immunity by vaccines is key for the rational development of novel vaccines and to optimize vaccine efficacy. Within the ADITEC work package “Molecular signatures of immunity and immunogenicity,” the aim was to identify key molecular mechanisms of innate and adaptive immunity following vaccination and to uncover correlates of protection at the transcriptomic, proteomic and cellular level.

Transcriptomic profiling identified IFN-inducible gene *FCGR1A* as one of the most robust and consistent single biomarkers that could serve as part of reliable predictive signatures discriminating active TB disease from latent infection, identifying individuals at risk of developing TB, and monitoring TB treatment response kinetics. Such signatures are useful in stratifying risk groups for preventive treatment or clinical vaccine testing, as well as minimizing treatment duration and preventing possible *de novo* drug resistance, independent of age, immune status and genetic background. By contrast, when comparing transcriptome profiles between infants vaccinated with parental or recombinant BCG VPM1002 to unravel the mechanisms underlying superior protection against TB by VPM1002, differences at the single gene level could not be detected. Therefore, a new bioinformatics tool (tmod) was used to calculate enrichments in genes, uncovering more pronounced changes in T-cell activation and myeloid/monocyte-associated genes in the VPM1002 vaccinated group. Complementing the work on transcriptomics, HDX–MS was used to determine quantitative proteomic profiles and was successfully applied to define functionally active epitopes and antibodies in polyclonal sera following vaccination for various bacterial vaccines.

At the cellular level, early expansion of vaccine-specific CD4+ Tfh cells in peripheral blood was found to predict long term persistence of neutralizing antibodies against influenza virus, suggesting this to be a possible biomarker of protective vaccine efficacy. In addition, it was observed that vaccine-induced human CD4+ T-cells are functionally heterogeneous and comprise not only clones polarized toward a single fate but also clones that fit the one cell-multiple fates model of CD4 T-cell differentiation. This suggests that inducing broader range of effector and memory T-cells with broad and different functional properties would benefit the host by expanding the number of options to respond to pathogens. Parameters affecting the ability of leukocytes to properly migrate to the site of vaccination and mount an effective immune response were also investigated. Interestingly, persistent immune activation caused impaired T-cell trafficking at mucosal sites, which was due to alterations in the cytoskeleton machinery. Pharmacological intervention acting on the cytoskeleton could restore a proper response to chemokines, calling for novel vaccination strategies in frail individuals. Finally, PTX3 was identified as a molecule produced by the innate immune system in response to pro-inflammatory mediators and was shown to be involved in the innate defense against recognized pathogens. PTX3 functions as an endogenous adjuvant, bridging the humoral arms of the innate and adaptive immune systems.

In summary, within the ADITEC work package “Molecular signatures of immunity and immunogenicity,” dissecting the human innate and adaptive immune response to vaccination and infection identified not only novel biomarker signatures at the transcriptomic, proteomic, and cellular level that could serve as potential correlates of risk and of protection but also uncovered novel markers and mechanisms underlying protective immunity. Validating and further expanding on these findings will advance our understanding of protective immunity, which is a key step toward the development of effective novel vaccines.

## Author Contributions

All the authors have contributed a section to the manuscript, based on their research in the EC FP7 ADITEC project.

## Conflict of Interest Statement

The authors declare that the research was conducted in the absence of any commercial or financial relationships that could be construed as a potential conflict of interest.

## Footnotes

^1^Gebremicael GKD, Quinten E, Alemayehu Y, Gebreegziaxier A, Belay Y, et al. Host gene expression kinetics during treatment of tuberculosis in HIV-coinfected individuals is independent of HAART therapy (Forthcoming).

^2^Garlanda C, Bottazzi B, Magrini E, Inforzato A, Mantovani A. PTX3, a key component of humoral innate immunity at the interface between defense and tissue remodeling. *Physiol Rev* (Forthcoming).
